# Comparison of Conventional and Fiber‐Reinforced Restorative Protocols Using Standardized Three‐Dimensional Printed Cracked Tooth Models: An In Vitro Study

**DOI:** 10.1155/ijod/9975701

**Published:** 2026-10-25

**Authors:** Isabella Sousa Corrêa, Kênia Maria Pereira Soares de Toubes, Alberto Nogueira da Gama Antunes, Paulo Isaías Seraidarian, Stephanie Quadros Tonelli, Daniel Sousa Pardini, Gilberto Antônio Borges, Frank Ferreira Silveira

**Affiliations:** ^1^ Department of Dentistry Pontifical Catholic University of Minas Gerais (PUCMinas) Belo Horizonte Brazil; ^2^ Department of Dentistry Universidade de Uberaba (Uniube) Uberaba Brazil uniube.br

**Keywords:** 3D-printed models, composite resin, cracked teeth, fiber-reinforced composites, fracture resistance, polyethylene fiber

## Abstract

**Objective:**

To compare the fracture resistance and failure patterns of different restorative protocols in standardized three‐dimensional (3D)‐printed cracked molars.

**Methods:**

Sixty standardized mandibular molar models were fabricated from a single digital design using 3D printing technology and randomly allocated into five experimental groups (*n* = 12): sound teeth, cracked teeth (CT), CT with mesio‐occluso‐distal (MOD) cavity preparation, MOD cavities restored with conventional composite resin, and MOD cavities restored with a polyethylene fiber‐reinforced composite protocol. Fracture resistance was evaluated by static compressive loading. Failure patterns were classified according to predefined criteria. Data were analyzed using the Kruskal–Wallis test followed by Dunn’s post hoc test with Bonferroni adjustment (*α* = 0.05).

**Results:**

Significant differences in fracture resistance were observed among the experimental groups (*p* < 0.001). The polyethylene fiber‐reinforced composite group exhibited the highest fracture resistance (1288 ± 345 N), whereas the unrestored MOD group showed the lowest values (187 ± 63 N). The fiber‐reinforced and conventional composite groups did not differ significantly in fracture resistance (Bonferroni‐adjusted *p* = 1.000). Failure patterns showed a descriptively heterogeneous distribution within the fiber‐reinforced restorative protocol.

**Conclusions:**

Within the limitations of this in vitro study, the fiber‐reinforced restorative protocol demonstrated fracture resistance comparable to sound teeth and conventional composite restorations under static loading. The observed failure patterns differed descriptively among restorative protocols. These findings provide preliminary biomechanical evidence regarding the performance of different restorative strategies in a standardized 3D‐printed model.

## 1. Introduction

Cracked teeth (CT) are defined as incomplete fracture lines of variable depth and orientation that propagate through dental tissues, frequently involving the marginal ridges and occlusal surface, with possible extension toward the pulp or periodontal ligament [1, 2]. The development of these defects is generally progressive and has been associated with occlusal overload, parafunctional habits, restorative procedures, caries, and age‐related structural changes [3–5].

Diagnosing CT remains a considerable clinical challenge because the signs and symptoms are often subtle, intermittent, and nonspecific [2, 6]. Although pulpal vitality may initially be preserved, bacterial penetration along crack lines may eventually result in irreversible pulpitis or pulpal necrosis [7, 8]. Consequently, early diagnosis followed by timely restorative intervention is essential to stabilize the remaining tooth structure and improve its biomechanical behavior before further structural deterioration occurs [9, 10].

Despite the clinical importance of CT, there is still no consensus regarding the optimal restorative management of these teeth. Contemporary restorative approaches seek to preserve the remaining dental structure while improving the biomechanical behavior of structurally compromised teeth under functional loading [4, 11]. However, restorative procedures themselves may substantially influence the stress distribution within the tooth. Mesio‐occluso‐distal (MOD) cavity preparations, for example, significantly reduce tooth stiffness because of marginal ridge removal and the consequent increase in cuspal deflection [12, 13]. This structural weakening increases the stress concentration within the crown and may predispose the tooth to catastrophic fracture under occlusal loading [14].

Within the contemporary biomimetic restorative philosophy, preservation of sound dental tissues while restoring biomechanical integrity has become one of the principal objectives of restorative treatment. Adhesive restorative strategies capable of redistributing functional stresses without excessive removal of healthy tissues may contribute to a more conservative management of structurally compromised teeth. Direct composite resin restorations have therefore been proposed as minimally invasive restorative alternatives capable of partially restoring tooth stiffness and improving stress distribution within the tooth–restoration complex [15]. Nevertheless, their biomechanical performance remains strongly influenced by cavity geometry, remaining tooth structure, and material properties [12, 14].

To overcome these limitations, reinforcement strategies based on fiber incorporation into composite restorations have received increasing attention. Polyethylene fibers exhibit high tensile strength, flexibility, and favorable stress distribution characteristics, allowing them to function as reinforcing elements within the composite matrix [16–18]. Their reinforcing effect has been attributed to crack‐bridging and stress redistribution mechanins, which reduce the stress concentration at critical regions within the restoration. When strategically positioned across the occlusal portion of the cavity, connecting the buccal and lingual walls, polyethylene fibers may reduce cuspal deflection, improve fracture resistance, and modify the failure behavior [19]. Furthermore, recent systematic reviews and meta‐analyses have reported favorable fracture resistance of fiber‐reinforced composite restorations compared with conventional composite restorations [20, 21].

One of the major limitations of laboratory investigations involving CT is the difficulty in reproducing cracks with a standardized morphology. Naturally occurring cracks exhibit substantial variability regarding location, orientation, depth, and extension, compromising experimental standardization and potentially affecting biomechanical outcomes [6]. The three‐dimensional (3D) printing technology has emerged as a promising alternative for producing highly standardized experimental models with controlled crack geometry and excellent reproducibility [22]. Although these printed models do not reproduce the hierarchical structure or the mechanical properties of enamel and dentin, they provide standardized polymeric substrates that minimize specimen variability and allow controlled comparisons among restorative strategies. Consequently, they represent a reproducible experimental platform for preliminary biomechanical investigations while avoiding the inherent heterogeneity of extracted human teeth.

Therefore, the aim of this in vitro study was to compare the fracture resistance and failure patterns of different restorative protocols in standardized 3D‐printed cracked molars following MOD cavity preparation.

The null hypotheses were that the restorative protocols would not influence (1) fracture resistance or (2) failure patterns under the experimental conditions evaluated.

## 2. Materials and Methods

### 2.1. Study Design

This in vitro experimental study was designed to compare the biomechanical performance of different restorative strategies for standardized cracked molars using a controlled 3D‐printed polymeric model. Sixty specimens were allocated into five experimental groups (*n* = 12) according to the presence of a crack, cavity preparation, and restorative protocol.

The primary outcome was fracture resistance under static compressive loading, while the secondary outcome was the distribution of failure patterns after mechanical testing. The use of standardized 3D‐printed specimens was intended to minimize variations in tooth morphology, crack geometry, and cavity configuration, thereby improving experimental reproducibility and allowing direct comparison among restorative protocols.

It should be emphasized that the present model was not designed to reproduce the exact mechanical behavior of natural enamel and dentin. Rather, it represents a standardized polymeric experimental model developed to investigate the relative biomechanical behavior of different restorative approaches under controlled laboratory conditions. Therefore, the results should be interpreted within the limitations inherent to static in vitro testing and should not be directly extrapolated to the clinical performance of naturally CT.

The sample size (*n* = 12 specimens per group) was defined based on previous in vitro studies evaluating fracture resistance using comparable experimental designs. Although no formal a priori power analysis was performed, the selected sample size is consistent with that commonly reported in the restorative dentistry literature.

### 2.2. Ethical and Environmental Considerations

This in vitro study was conducted exclusively using standardized 3D‐printed polymeric specimens and did not involve human participants, animals, or biological tissues. Therefore, approval by an Institutional Review Board or Ethics Committee was not required according to institutional regulations. The use of standardized printed specimens reduced the need for extracted human teeth, contributing to greater experimental standardization while minimizing ethical concerns associated with the acquisition and storage of biological specimens. All laboratory procedures and disposal of polymeric materials were performed in accordance with institutional laboratory safety and environmental regulations.

### 2.3. Specimen Fabrication and Standardization

Three artificial mandibular molars (IM do Brasil, São Paulo, Brazil) were used as master models for specimen fabrication. One tooth was intact, whereas two teeth contained standardized mesiodistal crack lines and were radiopaque under radiographic examination, allowing radiographic verification of the crack configuration before digitalization.

The master teeth were digitized using an intraoral CAD/CAM scanner (3Shape, Copenhagen, Denmark), generating high‐resolution 3D digital models. The digital files were refined using CAD software (Exocad GmbH, Darmstadt, Germany) and subsequently reproduced by additive manufacturing using a stereolithographic 3D printer (Anycubic Photon Mono x6 K, Shenzhen, China) and a photopolymer printing resin (Proton, Belo Horizonte, MG, Brazil). This workflow ensured that all specimens presented identical external anatomy and internal geometric characteristics.

To standardize the crack geometry, the original crack was digitally refined in CAD software according to radiographic measurements obtained from the reference specimen. The standardized crack was designed as an open *V*‐shaped superficial notch with a depth of 2.5 ± 0.2 mm and a width of 0.1 ± 0.02 mm, oriented vestibulo–lingually and located mesially, crossing the marginal ridge. The crack was limited to the crown and did not involve the pulp chamber. This standardized geometry was reproduced identically in all cracked specimens (Figure 1).

**Figure 1:**
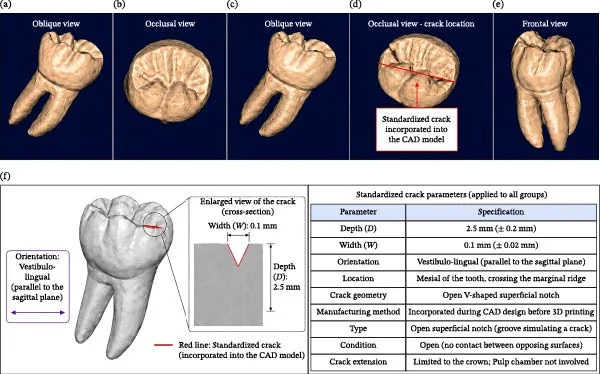
Standardized three‐dimensional (3D)‐printed mandibular molar prototype and crack design. (a) Oblique view of the intact prototype, (b) occlusal view, (c) oblique view of the cracked prototype, (d) occlusal view showing the location of the standardized crack (red line) incorporated into the CAD model, (e) frontal view, and (f) schematic representation of the standardized crack geometry and the standardized geometric parameters used for all specimens.

After fabrication of the cracked master model, a standardized MOD cavity preparation was performed on the reference tooth. The prepared specimen was rescanned using the same CAD/CAM system, generating a second digital model from which all specimens requiring cavity preparation were printed. Consequently, all specimens in the restored groups presented identical cavity dimensions and morphology.

The use of 3D‐printed specimens was intended to minimize specimen‐to‐specimen variability in tooth morphology, crack configuration, and cavity preparation, thereby improving experimental reproducibility. It should be emphasized that the printed teeth represent standardized polymeric experimental models and were not intended to reproduce the exact mechanical properties of natural enamel and dentin. Instead, the model was designed to provide controlled geometric standardization for biomechanical comparison of restorative strategies under laboratory conditions.

The design of the standardized 3D‐printed mandibular molar prototype and the geometry of the standardized crack incorporated into the CAD model are illustrated in Figure 1.

### 2.4. Experimental Groups

A total of 60 standardized 3D‐printed mandibular molars were assigned to five experimental groups (*n* = 12) according to the crack condition, cavity preparation, and restorative protocol. The groups were designed to simulate progressive structural conditions, ranging from intact teeth to CT restored using different adhesive strategies.

• Group I (sound teeth): Intact specimens without crack simulation or cavity preparation.
• Group II (CT): Specimens presenting a standardized mesiodistal incomplete crack without cavity preparation.
• Group III (CT with MOD preparation): Cracked specimens receiving a standardized MOD cavity preparation without restoration.
• Group IV (composite resin restoration): Cracked specimens with standardized MOD preparations were restored using a conventional adhesive protocol and direct composite resin.
• Group V (polyethylene fiber‐reinforced composite restoration): Cracked specimens with standardized MOD preparations restored using the same adhesive protocol as Group IV, with additional reinforcement using a polyethylene fiber ribbon (Ribbond, Ribbond Inc., Seattle, WA, USA) embedded within the composite restoration.

Because all specimens were fabricated from the same standardized digital model, the only experimental variables among the groups were the presence of the crack, cavity preparation, and restorative protocol.

### 2.5. Cavity Preparation

A standardized MOD cavity preparation was performed on the cracked master tooth according to the conservative cavity preparation principles described by Mondelli et al. [12]. Preparations were carried out by a single calibrated operator using a #245 carbide bur (KG Sorensen, São Paulo, Brazil) under copious water cooling.

The cavity design included an axial wall depth of approximately 1.2 mm, with proximal boxes extending to the cemento‐enamel junction to encompass the entire standardized crack. The buccal and lingual walls of the proximal boxes were prepared with a slight occlusal convergence, while the pulpal floor was flat and perpendicular to the long axis of the tooth. Internal line angles were rounded using a #4138 diamond bur (KG Sorensen, São Paulo, Brazil), and the occlusal isthmus width corresponded to approximately one‐quarter of the intercuspal distance. The cavity preparation was designed to completely include the standardized crack while preserving the integrity of the simulated pulp chamber.

After cavity preparation, the master tooth was rescanned using the same CAD/CAM system (3Shape, Copenhagen, Denmark). The resulting digital model was subsequently used to fabricate all specimens assigned to Groups III, IV, and V by 3D printing, ensuring identical cavity geometry and highly standardized specimen production for biomechanical testing.

### 2.6. Restorative Procedures

The restorative procedures were performed in Groups IV and V by a single calibrated operator, following a standardized adhesive protocol. Materials used during specimen fabrication and restorative procedures are presented in Table 1.

**Table 1:** Commercial materials used for fabrication of the standardized 3D‐printed specimens and restorative procedures, including manufacturers, principal chemical composition, intended application, and batch numbers.

Material	Manufacturer	Main composition	Clinical application
Proton 3D Printing Resin	Proton	Photopolymer resin	Fabrication of standardized 3D‐printed specimens
Clearfil SE Bond Primer	Kuraray Noritake Dental Inc., Tokyo, Japan	10‐MDP, HEMA, hydrophilic dimethacrylate	Adhesive primer
Clearfil SE Bond Adhesive	Kuraray Noritake Dental Inc., Tokyo, Japan	10‐MDP, Bis‐GMA, HEMA, hydrophobic dimethacrylate	Adhesive bonding agent
Silane	Angelus, Londrina, PR, Brazil	Organosilane coupling agent	Silanization of the fiber
Forma	Ultradent Products Inc., South Jordan, UT, USA	Nanohybrid composite resin (Bis‐GMA, UDMA, inorganic fillers)	Direct restoration
GrandioSO Heavy Flow	VOCO GmbH, Cuxhaven, Germany	Flowable nanohybrid composite resin (glass‐ceramic fillers, Bis‐GMA/UDMA matrix)	Fiber embedding and adaptation
Ribbond	Ribbond Inc., Seattle, WA, USA	Ultra‐high‐molecular‐weight polyethylene (UHMWPE) fiber	Reinforcement of composite restoration

Abbreviations: Bis‐GMA, bisphenol A‐glycidyl methacrylate; TEGDMA, triethylene glycol dimethacrylate; UDMA, urethane dimethacrylate.

Initially, Clearfil SE Bond Primer (Kuraray Noritake Dental Inc., Tokyo, Japan) was actively applied to all cavity surfaces for 20 s and gently air‐dried according to the manufacturer’s instructions. For Group V, the polyethylene fiber ribbon was silanized with a silane coupling agent (Angelus, Londrina, PR, Brazil), followed by warm‐air evaporation for 2 min. Clearfil SE Bond adhesive (Kuraray Noritake Dental Inc., Tokyo, Japan) was then applied, the excess was carefully removed with absorbent paper, and the adhesive was light‐cured for 20 s using an LED curing unit (VALO Grand, Ultradent Products Inc., South Jordan, UT, USA) operating in the standard curing mode (~1000 mW/cm^2^, according to the manufacturer’s specifications). During all curing procedures, the light guide tip was positioned perpendicularly and as close as possible to the restoration surface.

In Group IV, the cavities were restored with a nanohybrid composite resin (Forma, Ultradent Products Inc., South Jordan, UT, USA) using the incremental technique. Each increment, not exceeding 2 mm in thickness, was light‐cured for 30 s, followed by a final curing cycle of 40 s.

In Group V, after completion of the adhesive protocol, the cavity floor was initially covered with a thin layer of flowable composite resin (GrandioSO Heavy Flow, VOCO GmbH, Cuxhaven, Germany). A polyethylene fiber ribbon (Ribbond, Ribbond Inc., Seattle, WA, USA), measuring 8 mm in length and 2 mm in width, was uniformly impregnated with the adhesive system and further coated with the same flowable composite resin before placement. The fiber was positioned in a buccolingual direction, connecting the cavity walls and remaining completely embedded within the flowable composite. After the removal of excess material, the assembly was light‐cured for 30 s. The cavity was then restored with the same incremental composite resin technique used in Group IV, followed by a final light‐curing cycle of 40 s.

The main chemical compositions were obtained from the manufacturers’ technical information. Batch numbers correspond to the materials used in the present study.

### 2.7. Storage Conditions

After completion of the restorative procedures, all specimens were stored individually in distilled water at 37°C for 24 h before mechanical testing. This storage period was adopted to allow post‐polymerization stabilization of the restorative materials under standardized conditions before fracture‐resistance testing.

### 2.8. Fracture Resistance Test

The root portion of each specimen was coated with utility wax and embedded in self‐curing acrylic resin (JET Clássico, São Paulo, SP, Brazil) within polyvinyl chloride (PVC) cylinders up to the cemento‐enamel junction. A polyether impression material (Impregum Soft, 3M ESPE, St. Paul, MN, USA) was used to simulate the periodontal ligament. A custom positioning device was employed to ensure standardized specimen alignment during embedding and mechanical testing (Figure 2).

**Figure 2:**
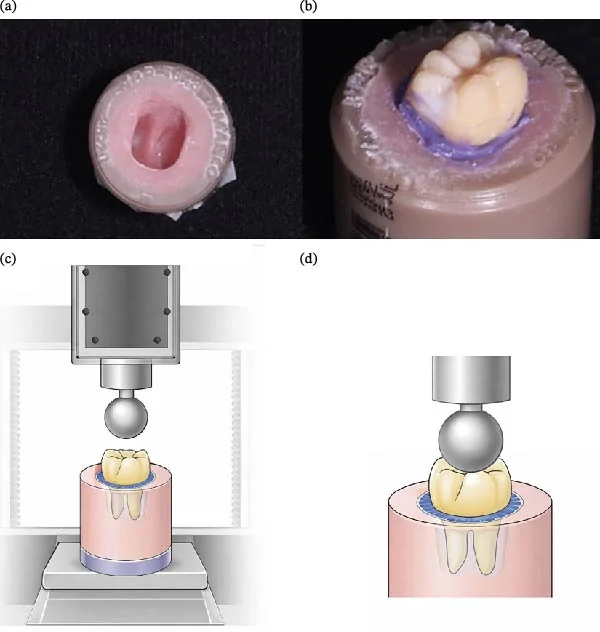
Experimental setup for fracture resistance testing. (a) Polyvinyl chloride (PVC) cylinder used for specimen embedding, (b) standardized 3D‐printed mandibular molar embedded in self‐curing acrylic resin with a simulated periodontal ligament reproduced using polyether impression material, (c) schematic illustration of the specimen positioned in the universal testing machine, and (d) detailed view of axial compressive loading using a spherical stainless‐steel indenter positioned perpendicular to the occlusal surface. Compressive loading was applied simultaneously to the buccal and lingual cusps at a crosshead speed of 1.0 mm/min until catastrophic fracture occurred.

Fracture resistance was evaluated using a universal testing machine (EMIC DL Series, Instron, Norwood, MA, USA) equipped with a 500 kgf load cell. A compressive load was applied using a spherical stainless‐steel indenter positioned perpendicular to the occlusal surface at a crosshead speed of 1.0 mm/min until catastrophic fracture occurred. The load was applied simultaneously to the buccal and lingual cusps, and the maximum fracture load was recorded in Newton (N).

### 2.9. Failure Mode Analysis

Following fracture resistance testing, all specimens were visually inspected and examined under a stereomicroscope at × 10 magnification to characterize the fracture patterns. Fractures were classified in four categories:

• Type I: Cuspal fracture restricted to the tooth structure without the involvement of the restorative material.
• Type II: Adhesive failure at the tooth–restoration interface.
• Type IIIA: Cohesive failure restricted to the composite resin.
• Type IIIB: Combined cohesive failure involving both the composite resin and the polyethylene fiber reinforcement.

Failure modes were classified according to predefined criteria proposed by Schmidt et al. to ensure consistency in specimen evaluation (Figure 3).

**Figure 3:**
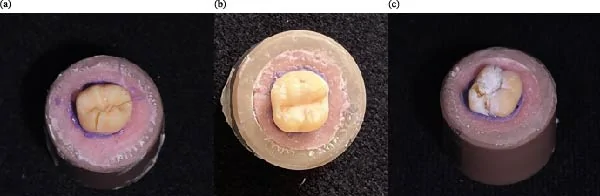
Representative fracture patterns observed after fracture resistance testing. (a) Type I fracture restricted to the tooth structure without involvement of the restorative material. (b) Type IIIA cohesive failure restricted to the composite resin. (c) Type IIIB combined cohesive failure involving both the composite resin and the polyethylene fiber reinforcement (Ribbond).

### 2.10. Statistical Analysis

Statistical analyses were performed using the IBM SPSS Statistics software (version 25.0; IBM Corp., Armonk, NY, USA). Fracture resistance values were initially assessed for normality using the Kolmogorov–Smirnov test. As the data did not follow a normal distribution, nonparametric statistical methods were adopted.

Intergroup comparisons of fracture resistance were performed using the Kruskal–Wallis test, followed by the Dunn’s post hoc test for pairwise multiple comparisons when significant differences were detected. The significance level was set at *α* = 0.05.

The significance level was set at *α* = 0.05. The failure mode distributions were summarized using absolute frequencies and percentages. Because some failure categories were treatment‐dependent, particularly Type IIIB, and several categories had low frequencies, failure patterns were interpreted descriptively without inferential statistical comparison.

## 3. Results

### 3.1. Fracture Resistance

Fracture resistance differed significantly among the experimental groups (Kruskal–Wallis test, *H* = 45.0, df = 4, *p* < 0.001, *ε* ^2^ = 0.776). The polyethylene fiber‐reinforced composite restoration group exhibited the highest fracture resistance values (1288.00 ± 345.13 N), whereas the CT with the MOD preparation group presented the lowest values (187.42 ± 63.20 N). Sound teeth and CT without cavity preparation showed intermediate fracture resistance values and did not differ significantly from each other. The large effect size (*ε* ^2^ = 0.776) indicates that the experimental condition exerted a substantial influence on the fracture resistance.

Descriptive statistics, including mean ± standard deviation, median, interquartile range (IQR), minimum–maximum values, and statistical groupings obtained from Dunn’s post hoc test, are presented in Table 2.

**Table 2:** Fracture resistance of the experimental groups.

Experimental group	*n*	Mean ± SD (*N*)	Median (IQR) (*N*)	Range (min–max) (*N*)	Statistical grouping^b^
Sound teeth	12	771.42 ± 160.12	750.0 (717.8–828.5)	475–1117	B
Cracked teeth	11^a^	631.49 ± 168.38	561.0 (515.5–782.7)	386–880	B
Cracked teeth with MOD preparation	12	187.42 ± 63.20	197.0 (142.5–226.5)	57–268	C
Composite resin restoration	12	1035.58 ± 274.25	1071.0 (880.8–1199.5)	585–1547	AB
Polyethylene fiber‐reinforced composite restoration	12	1288.00 ± 345.13	1281.0 (1112.0–1410.3)	824–2007	A

*Note*: Fracture resistance was compared using the Kruskal–Wallis test (*H* = 45.0, df = 4, *p* < 0.001, *ε* ^2^ = 0.776), followed by Dunn’s post hoc test with Bonferroni adjustment. Groups sharing at least one uppercase letter were not significantly different (*p* < 0.05). Median (IQR) values are presented because fracture resistance data did not meet the assumption of normality. Statistically significant differences were observed between sound teeth and cracked teeth with MOD preparation (*p* = 0.0118), sound teeth and polyethylene fiber‐reinforced composite restorations (*p* = 0.0403), cracked teeth and polyethylene fiber‐reinforced composite restorations (*p* = 0.0022), cracked teeth with MOD preparation and composite resin restorations (*p* < 0.001), and cracked teeth with MOD preparation and polyethylene fiber‐reinforced composite restorations (*p* < 0.001).

^a^One specimen from the cracked teeth group was excluded because of experimental failure during fracture testing (*n* = 11).

^b^Data are presented as mean ± standard deviation, median (interquartile range), and minimum–maximum values.

The fiber‐reinforced restorative protocol yielded the highest fracture resistance values; however, it did not differ significantly from the conventional composite restoration (Bonferroni‐adjusted *p* = 1.000. The exact Bonferroni‐adjusted *p*‐values obtained from Dunn’s post hoc pairwise comparisons are presented in Table S1.

### 3.2. Failure Mode Analysis

The distribution of failure modes varied descriptively among the experimental groups (Table 3 and Figure 4). Sound teeth, CT, and unrestored MOD‐prepared teeth exhibited exclusively Type I failures. In the composite resin restoration group, cohesive failures restricted to the restorative material (Type IIIA) predominated, accounting for 83.3% of the specimens, whereas adhesive failures (Type II) were observed in 16.7%.

**Table 3:** Distribution of fracture failure modes according to the experimental groups.

Experimental group	Type I *n* (%)	Type II *n* (%)	Type IIIA *n* (%)	Type IIIB *n* (%)	Excluded due to experimental failure
Sound teeth	12 (100)	0	0	0	0
Cracked teeth	11 (91.7)	0	0	0	1 (8.3)
Cracked teeth with MOD preparation	12 (100)	0	0	0	0
Composite resin restoration	0	2 (16.7)	10 (83.3)	0	0
Polyethylene fiber‐reinforced composite restoration	5 (41.7)	0	3 (25.0)	4 (33.3)	0

*Note*: One specimen from the cracked teeth group was excluded because of experimental failure during fracture testing (*n* = 11).

**Figure 4:**
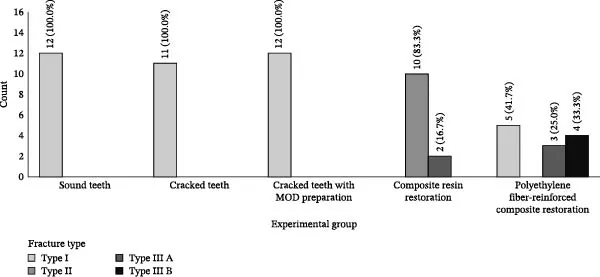
Distribution of failure modes according to the experimental groups. Stacked bar charts illustrate the percentage of specimens exhibiting each failure mode in the five experimental groups. The absolute number of specimens (*n*) is displayed within each bar segment. One specimen from the cracked teeth group was excluded because of experimental failure during fracture resistance testing (*n* = 11).

In contrast, the polyethylene fiber‐reinforced composite restoration group exhibited a more heterogeneous distribution of failure modes, including Type I (41.7%), Type IIIA (25.0%), and Type IIIB (33.3%) failures. Overall, the polyethylene fiber‐reinforced composite restoration group demonstrated a descriptively different distribution of failure patterns compared with those of the other experimental groups under the conditions evaluated.

A descriptive summary of the observed failure modes is provided in Table 3, and their distribution across the experimental groups is illustrated in Figure 4.

## 4. Discussion

The present study evaluated the fracture resistance and failure behavior of different restorative protocols in a standardized 3D‐printed model of CT. The fiber‐reinforced restorative protocol yielded the highest fracture resistance values among the restorative strategies evaluated and showed values comparable to those of sound teeth. However, no statistically significant difference was observed between the fiber‐reinforced and conventional composite restoration groups (Bonferroni‐adjusted *p* = 1.000). Therefore, these findings should not be interpreted as evidence of an independent reinforcing effect of polyethylene fiber. Rather, they reflect the overall biomechanical performance of the restorative protocol evaluated under standardized static in vitro conditions. These observations are consistent with previous laboratory investigations and systematic reviews reporting favorable biomechanical performance of fiber‐reinforced restorative approaches [17, 21–23].

Comparison among the experimental groups clearly demonstrated the detrimental effect of MOD cavity preparation on the biomechanical behavior of the specimens. Teeth subjected exclusively to MOD cavity preparation without restoration exhibited the lowest fracture resistance values, corroborating the well‐established relationship between marginal ridge removal and the loss of structural stiffness [10, 12–14]. Although the present study did not evaluate crack propagation directly, these biomechanical changes provide a plausible explanation for the marked reduction in fracture resistance observed in the unrestored MOD group. Collectively, these findings reinforce the importance of preserving a sound tooth structure whenever possible and support minimally invasive restorative approaches aimed at maintaining the biomechanical integrity of compromised teeth.

Interestingly, fracture resistance values observed in sound teeth and CT without cavity preparation did not differ significantly. This finding suggests that, within the limitations of the present standardized experimental model, the simulated crack alone did not substantially reduce the fracture resistance under static compressive loading. However, this observation should not be interpreted as evidence that cracks are clinically innocuous since the present study did not evaluate cyclic fatigue, thermomechanical aging, or crack propagation. In contrast, naturally CT are continuously exposed to cyclic occlusal loading, thermal fluctuations, and the oral environment, all of which may contribute to progressive crack propagation over time [9, 10, 24]. Therefore, the similarity observed between sound and CT in the present study should be interpreted as a characteristic of the experimental model rather than as a direct representation of the clinical behavior of naturally CT.

Both restorative protocols improved fracture resistance compared with that of unrestored MOD preparations. Although the fiber‐reinforced restorative protocol exhibited the highest fracture resistance values, these values were not statistically different from those obtained with conventional composite restoration. Therefore, the present findings should be interpreted as a comparison of restorative protocols rather than as evidence of an independent reinforcing effect of the fiber‐reinforced restorative protocol. This behavior is consistent with the ability of ultra‐high‐molecular‐weight polyethylene (UHMWPE) fibers to redistribute functional stresses and reduce stress concentration within the tooth–restoration complex. Previous laboratory studies have reported similar reinforcing effects, supporting the crack‐bridging mechanisms as a plausible explanation for the improved fracture resistance observed in the present study [17–19].

The present findings should be interpreted within the broader context of biomimetic restorative dentistry. Similar biomechanical improvements have been reported for indirect adhesive restorations with cuspal coverage and short‐fiber‐reinforced composites. Although these approaches differ in composition and clinical indication, they share the common objective of preserving the tooth structure while improving biomechanical performance. Therefore, polyethylene fiber reinforcement should be regarded as one promising biomimetic strategy rather than a universally superior restorative approach [21].

Failure mode analysis provided complementary information regarding the biomechanical behavior of the restored specimens following fracture testing. Sound teeth, CT, and unrestored MOD‐prepared specimens exhibited exclusively Type I failures, whereas the restorative groups demonstrated distinct failure patterns. Conventional composite restorations were predominantly associated with cohesive failures within the restorative material (Type IIIA), whereas polyethylene fiber‐reinforced restorations showed a more heterogeneous distribution, including Type I, Type IIIA, and Type IIIB failures. The observed distribution may reflect differences in the overall restorative protocols and their influence on load transfer within the tooth–restoration complex; however, these findings should be interpreted descriptively because some failure categories were treatment‐dependent and occurred at low frequencies. Similar observations have been reported in previous laboratory studies evaluating polyethylene fiber‐reinforced composite restorations, in which fiber incorporation was associated with altered fracture patterns and improved biomechanical performance [25].

An important strength of this study is the use of standardized 3D‐printed specimens, which minimized anatomical variability and standardized the crack geometry among groups. Additive manufacturing provides a highly reproducible platform for comparative biomechanical investigations; however, currently available photopolymer resins do not reproduce the hierarchical structure or mechanical properties of enamel and dentin. Therefore, the present model should be interpreted as a standardized polymeric platform for comparative biomechanical evaluation rather than as a direct mechanical substitute for natural dental tissues [22]. Accordingly, these findings should be interpreted within the context of this standardized experimental model and subsequently validated using natural teeth and long‐term aging protocols.

Contemporary management of cracked posterior teeth includes several conservative adhesive approaches aimed at preserving the tooth structure while improving biomechanical performance. Adhesively bonded cuspal coverage restorations, including onlays and overlays, and fiber‐reinforced techniques have been proposed to enhance the structural integrity and biomechanical performance of compromised teeth. In the present standardized static polymeric model, the fiber‐reinforced restorative protocol exhibited the highest fracture resistance values among the restorative strategies evaluated. These findings suggest that different restorative approaches may provide complementary biomechanical advantages depending on the clinical objective [4, 10, 15, 17, 24, 25].

Some limitations of the present study should be considered. Immediate static fracture testing was intentionally used to provide a standardized baseline for comparing the restorative protocols while minimizing additional experimental variables. However, this approach does not reproduce the cumulative effects of thermal fluctuations, cyclic loading, hydrolytic aging, or fatigue‐related crack propagation that contribute to the clinical progression of CT. Moreover, although the standardized 3D‐printed polymeric model reduced specimen variability, it cannot fully reproduce the structural complexity or mechanical behavior of natural enamel and dentin, and additive manufacturing parameters may also influence their mechanical performance. Therefore, the present findings should be interpreted within the limitations of this standardized static model. Future studies should incorporate thermocycling, cyclic fatigue loading, and validation using natural teeth to better simulate clinical conditions.

Overall, the present findings should be interpreted as representing the biomechanical performance of restorative strategies within a highly standardized laboratory model rather than the direct clinical behavior of naturally CT. Future studies should incorporate thermomechanical aging, cyclic fatigue loading, and validation using natural teeth or biomimetic substrates to further establish the long‐term biomechanical performance and clinical relevance of these restorative approaches.

## 5. Conclusion

Within the limitations of this in vitro study, the fiber‐reinforced restorative protocol demonstrated fracture resistance comparable to that of sound teeth and conventional composite restorations under static loading. These findings provide preliminary biomechanical evidence regarding the performance of different restorative protocols in a standardized 3D‐printed model. Further studies incorporating thermomechanical aging, cyclic fatigue, and natural teeth are required before clinical extrapolation.

## Author Contributions

**Isabella Sousa Corrêa**: methodology, investigation, writing – review and editing. **Kênia Maria Pereira Soares de Toubes**: conceptualization, methodology, investigation, formal analysis, writing – review and editing. **Alberto Nogueira da Gama Antunes**: methodology, formal analysis, investigation. **Paulo Isaías Seraidarian**: conceptualization, writing – review and editing. **Stephanie Quadros Tonelli, Daniel Sousa Pardini, and Gilberto Antônio Borges**: writing – review and editing. **Frank Ferreira Silveira**: conceptualization, supervision, project administration, writing – review and editing.

## Funding

No funding was received for this manuscript.

## Disclosure

The lead author, Frank Ferreira Silveira, stated that this manuscript is an honest, accurate, and transparent account of the study being reported; there are no important aspects of the study that have been omitted.

## Ethics Statement

As this investigation was conducted exclusively as an in vitro laboratory study using three‐dimensional printed tooth models, without the involvement of human participants, human biological tissues, identifiable patient information, or animals, Institutional Ethics Committee approval was not required.

## Conflicts of Interest

The authors declare no conflicts of interest.

## Supporting Information

Additional supporting information can be found online in the Supporting Information section.

## Supporting information


**Supporting Information** Table S1: Exact Bonferroni‐adjusted *p*‐values from Dunn’s post hoc pairwise comparisons of fracture resistance among the experimental groups.

## Data Availability

The authors confirm that the data supporting the findings of this study are available within the article and its Supporting Information.
