# Supplementary material for: Comparison of Conventional and Fiber‐Reinforced Restorative Protocols Using Standardized Three‐Dimensional Printed Cracked Tooth Models: An In Vitro Study
Source: Int J Dent. 2026 Oct 25;2026:9975701. doi: 10.1155/ijod/9975701 (PMC13695836; doi:10.1155/ijod/9975701)
Supplement: Supplementary file 1 [file IJOD-2026-9975701-s001.docx]

Supplementary Table S1. Exact Bonferroni-adjusted p-values from Dunn's post hoc pairwise comparisons of fracture resistance among the experimental groups.

| **Pairwise comparison** | | **Adjusted p-value** |
| --- | --- | --- |
| Sound × Cracked | p = 1.000 | |
| Sound × MOD | p = 0.018 | |
| Sound × Composite | p = 1.000 | |
| Sound × Fiber-reinforced protocol | p = 0.090 | |
| Cracked × MOD | p = 0.391 | |
| Cracked × Composite | p = 0.136 | |
| Cracked × Fiber-reinforced protocol | p = 0.003 | |
| MOD × Composite | p < 0.001 | |
| MOD × Fiber-reinforced protocol | p < 0.001 | |
| Composite × Fiber-reinforced protocol | p = 1.000 | |

Pairwise comparisons were performed following the Kruskal–Wallis test. Adjusted p-values were calculated using the Bonferroni correction. Significant comparisons (p < 0.05) are indicated in bold. **Abbreviations:** ST, sound teeth; CT, cracked teeth; MOD, unrestored MOD cavity; CR, conventional composite restoration; FR, fiber-reinforced restorative protocol.
